# Low dose immunoglobulin g for treatment of severe sepsis and septic shock

**DOI:** 10.1186/2197-425X-3-S1-A433

**Published:** 2015-10-01

**Authors:** Y Iizuka, M Sanui, M Hayakawa, S Uchino, T Mayumi, D Kudo, S Saito, K Takimoto, K Yamakawa, Y Sasabuchi

**Affiliations:** Department of Anesthesiology and Critical Care Medicine, Jichi Medical University Saitama Medical Center, Saitama, Japan; Emergency and Critical Care Center, Hokkaido University Hospital, Sapporo, Japan; Intensive Care Unit, Department of Anesthesiology, Jikei University School of Medicine, Tokyo, Japan; Department of Emergency Medicine, University of Occupational and Environmental Health, Kitakyusyu, Japan; Division of Emergency and Critical Care Medicine, Tohoku University Graduate School of Medicine, Sendai, Japan; Department of Anesthesiology and Intensive Care Medicine, Osaka University, Osaka, Japan; Division of Molecular & Vascular Medicine, Beth Israel Deaconess Medical Center, Boston, MA USA; Department of Clinical Epidemiology and Health Economics, School of Public Health, The University of Tokyo, Tokyo, Japan

## Introduction

As an adjunctive treatment in sepsis, patients administered high-dose (0.9g/kg body weight) intravenous immunoglobulin G (IvIgG) did not have a significant survival benefit in a randomized control study (The SBITS study) (1). However, low-dose ivIgG (5g/day 3days) might have some utilities including earlier defervescence and improvements of clinical signs and symptoms (2).

## Objectives

In this retrospective cohort study of patients with severe sepsis and septic shock, we investigated whether low-dose IvIgG was associated with clinically important outcomes including ICU mortality and in-hospital mortality.

## Methods

This study is a preplanned retrospective analysis of a large database created by Japan SEPTIC DIC study conducted in 41 ICUs. This study investigated associations between sepsis-related coagulopathy, anticoagulation therapies, and clinical outcomes of 3195 adult patients with severe sepsis and septic shock admitted to ICUs in Japan from January 2011 through December 2013. To estimate associations between IvIgG administration and mortalities, multivariable logistic regression modeling and propensity score-based matching were used for analysis with SPSS version 22.

## Results

IvIgG was administered in 976 patients (30.5%). Patients administered ivIgG had significantly higher APACHE II scores (24.2 ± 8.8 vs. 22.7 ± 8.6, P < 0.001), and SOFA scores at admission (10.4 ± 4.0 vs. 9.1 ± 4.0, P < 0.001). ICU mortality was higher in patients with IvIgG (23.1% vs. 18.4%, P = 0.003), but IvIgG was not associated with ICU mortality after adjustment for cofactors (OR 1.121, 95%CI 0.879-1.430, P = 0.358). In-hospital mortality in patients receiving IvIgG was similar to those not receiving IvIgG (34.7% vs 31.9% P = 0.118). In a propensity score-matched analysis, both ICU mortality and in-hospital mortality were not different between the groups (22.4% vs. 19.1%, P = 0.088; and 33.7% vs. 31.8%, P = 0.388, respectively).

## Conclusions

In our retrospective analysis of a large cohort of severe sepsis and septic shock, administration of low-dose IvIgG as adjunctive therapy for patients with severe sepsis and septic shock was not associated with a reduction in ICU mortality and in-hospital mortality.
